# The role of pluripotency regulators in triple-negative breast cancer immune response

**DOI:** 10.3389/fgene.2026.1771872

**Published:** 2026-04-23

**Authors:** Carolina López-Santana, Fabio Mendez-Rivera, David A. Bernal-Estévez

**Affiliations:** Cell Therapy, Immunology and Clinical Oncology Research Group (GIIOC), Fundación Salud de Los Andes, Bogotá, Colombia

**Keywords:** breast cancer stem cells, immune response, pluripotency regulators, transcription factors, triple negative breast cancer

## Abstract

Triple-negative breast cancer (TNBC) is defined by the absence of estrogen, progesterone, and HER2 receptor expression. A critical challenge in managing TNBC is its high concentration of cancer stem cells (CSCs), which drives chemotherapy resistance and correlates with poor patient survival. In normal physiology, stem cell pluripotency and differentiation are governed by core transcription factors (such as Oct4, Sox2, Nanog, Klf4, and c-Myc) alongside key signaling networks, including the Notch, Wnt/β-catenin, and Sonic Hedgehog (Shh) pathways. During carcinogenesis, aberrant activation of these regulators in TNBC not only promotes the self-renewal of tumor cells but also actively facilitates immune evasion. Specifically, overexpressed pluripotency transcription factors enable cancer cells to downregulate antigen presentation molecules (e.g., MHC class I) and secrete immunomodulatory cytokines. Concurrently, dysregulated signaling, such as the Wnt/β-catenin pathway, inhibits dendritic cell maturation and recruits Myeloid-Derived Suppressor Cells (MDSCs) and regulatory T cells (Tregs) into the tumor microenvironment, thereby blunting the anti-tumor T cell response. This review examines the role of key pluripotency regulators in TNBC-mediated immune evasion, highlighting emerging immunotherapeutic strategies targeting these networks and summarizing current clinical research.

## 1 Introduction

Breast cancer remains a paramount global health problem, affecting millions of individuals worldwide. Among its various subtypes, triple-negative breast cancer (TNBC) represents a particularly complex clinical challenge. Accounting for approximately 15%–20% of all diagnosed breast cancers, TNBC is defined by the lack of estrogen receptor (ER), progesterone receptor (PR), and human epidermal growth factor receptor 2 (HER2) expression, typically confirmed via immunohistochemical staining (Yin et al., 2020; de Ruijter et al., 2011).

This molecular null phenotype renders traditional endocrine and HER2-targeted therapies ineffective, leaving systemic chemotherapy and, increasingly, immunotherapy as the primary therapeutic modalities (de Ruijter et al., 2011; Anders and Carey, 2008). While TNBC often exhibits initial sensitivity to cytotoxic chemotherapy, it is paradoxically associated with a high propensity for early recurrence, distant metastasis, and ultimately, a poorer prognosis compared to other breast cancer subtypes (Anders and Carey, 2008; Bianchini et al., 2016).

The aggressive clinical behavior and frequent therapy resistance observed in TNBC are heavily attributed to the tumor microenvironment’s high enrichment of cancer stem cells (CSCs) (Won and Spruck, 2020). Breast cancer stem cells (BCSCs) represent a distinct, dynamic subpopulation of neoplastic cells possessing the unique capacities for self-renewal and multi-lineage differentiation (Park et al., 2019). These properties are fundamental drivers of tumor initiation, sustained heterogeneous growth, metastatic dissemination, and evasion of conventional chemo- and radiotherapies (Valent et al., 2012; He et al., 2021). The clinical challenge of TNBC is largely defined by the imperative to effectively eradicate this elusive BCSC reservoir to prevent relapse.

In normal physiology, stem cell pluripotency and controlled differentiation are orchestrated by a precise network of intracellular signaling pathways—including Notch, Wnt/β-catenin, Sonic Hedgehog (Shh), and TGF-β/SMAD—alongside a core circuitry of transcription factors such as Oct4, Sox2, Nanog, Klf4, and c-Myc (Fultang et al., 2021). However, during carcinogenesis, TNBC cells frequently hijack these developmental networks. The aberrant overexpression of these pluripotency regulators drives a transcriptional reprogramming that sustains the self-renewal and malignant plasticity of tumor cells (Serrano Garcia et al., 2024).

Significantly, the reactivation of these embryonic programs extends beyond tumor growth; it actively modulates the tumor-immune landscape. This review will delve into the intricate interplay between key pluripotency regulatory transcription factors and the immune response in TNBC. We explore how the dysregulation of these factors and their associated signaling axes not only maintains the BCSC state but also equips these cells to evade immune surveillance and elimination. Furthermore, we examine the translational potential of targeting these pluripotency networks as a novel immunotherapeutic strategy, providing a comprehensive overview of the current clinical research landscape.

## 2 Characteristics of triple-negative breast cancer (TNBC)

TNBC is unequivocally defined by the concurrent absence of estrogen receptor (ER), progesterone receptor (PR), and human epidermal growth factor receptor 2 (HER2) expression. This triple-negative phenotype is the hallmark of TNBC and dictates the ineffectiveness of endocrine therapies targeting ER and PR, as well as HER2-directed agents like trastuzumab (Yin et al., 2020). Clinically, this diagnosis is routinely established through immunohistochemical (IHC) staining and, when necessary, Fluorescence *In Situ* Hybridization (FISH) to definitively confirm or exclude equivocal HER2 expression (de Ruijter et al., 2011). This well-defined molecular identity is consistently reported across numerous studies, underscoring its significance as a distinct and formidable breast cancer subtype. The primary clinical challenge in managing TNBC stems directly from this lack of conventional therapeutic targets.

Beyond its receptor status, the aggressive nature of TNBC is heavily driven by its profound cellular heterogeneity. Within the tumor bulk, only a discrete subpopulation of cells—known as cancer stem cells (CSCs) possesses the capacity to self-renew, proliferate, and seed new tumors. These cells were first functionally described in human breast cancer by Al-Hajj et al. in 2003, who demonstrated their ability to generate new tumors when xenografted into immunocompromised mice. Through the evaluation of cell surface markers, this tumorigenic population was identified by a CD44+/CD24−/low/Lineage^−^ phenotype (Al-Hajj et al., 2003). Further characterization has delineated tumorigenic from non-tumorigenic populations, the latter largely comprising the differentiated progeny of CSCs. Additionally, a broader category of cancer stem cell-like cells (CSCLCs) has been identified across various malignancies, including breast, brain, colon, and ovarian cancers, typically expressing surface markers such as CD133+, CD44+, CD24−, CD34+, CD29+, CD38−, CD166+, epithelial cell adhesion molecule (EpCAM), and Lin^−^ (Akbarzadeh et al., 2019).

**FIGURE 1:**
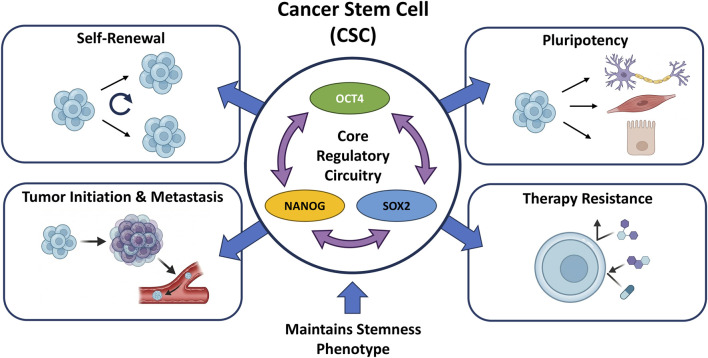
Core regulatory circuitry of cancer stem cells (CSCs) and their role in malignancy. The central elements show the key pluripotency transcription factors, specifically Oct4, Nanog, and Sox2, forming the core regulatory circuitry of the cancer stem cell (CSCs). Around the core regulatory of CSCs are shown the CSC properties as self-renewal, pluripotency, and their contribution to tumor initiation and metastasis and therapy resistance.

A critical and defining feature of the TNBC microenvironment is its notably high prevalence of these CSCs (Fultang et al., 2021). Compared to non-stem cancer cells, CSCs in TNBC exhibit enhanced tumorigenicity and a markedly greater capacity for metastasis. Furthermore, they are central to driving the extensive heterogeneity observed within TNBC tumors. Crucially, CSCs are implicated in the acquisition of the epithelial-mesenchymal transition (EMT) phenotype, a developmental program that enables malignant cells to detach from the primary tumor, invade local tissues, and disseminate to distant organs. The remarkable cellular plasticity of CSCs also allows them to undergo phenotypic shifts in response to varying therapeutic pressures (Guo and Han, 2023). Consequently, the enrichment of breast cancer stem cells (BCSCs) in TNBC is strongly correlated with adverse clinical outcomes, innate and acquired treatment resistance, and rapid tumor relapse. The persistence of these highly plastic CSCs—and the profound difficulty in eradicating them—represents a major hurdle in achieving long-term disease control (He et al., 2021; Fultang et al., 2021) (Figure 1).

Clinically, TNBC is characterized by an exceptionally aggressive disease course, frequently exhibiting rapid growth kinetics and a higher propensity for early dissemination compared to other invasive breast carcinomas. Patients with TNBC face a significantly higher risk of early cancer recurrence than those with hormone receptor-positive or HER2-positive subtypes (Bianchini et al., 2016). It is disproportionately likely to present with regional lymph node involvement or distant visceral metastases, limiting available treatment options and resulting in generally lower overall survival rates. Epidemiologically, the burden of TNBC is particularly pronounced in specific demographic and genetic cohorts; it is observed with significantly higher frequency in women younger than 40 years of age, in women of Black ethnicity (who face an incidence rate approximately twice as high as White women), and in carriers of germline BRCA1 mutations (Reis-Filho and Tutt, 2008). These intersecting demographic, genetic, and clinical features highlight the formidable underlying biology of this disease. Ultimately, to understand how these resilient CSC populations maintain their aggressive tumorigenic drive while concurrently shielding themselves from therapeutic and immunological eradication, it is imperative to examine the molecular pathways governing their plasticity.

## 3 Dysregulation and immune response modulation of stem cell signaling pathways in TNBC

**FIGURE 2:**
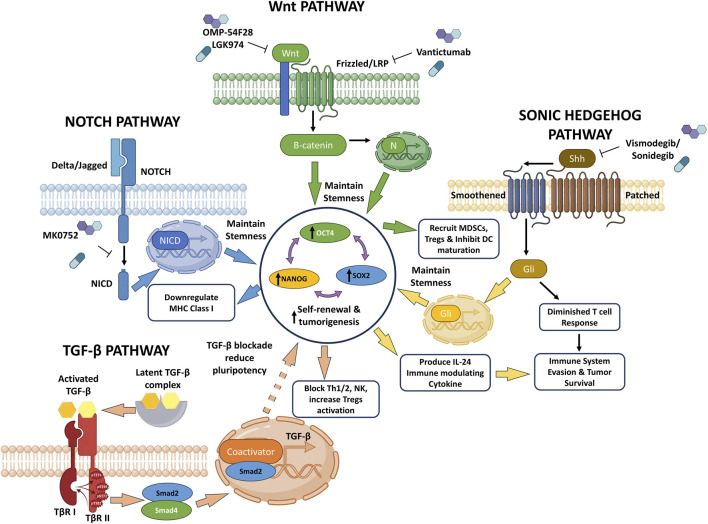
Detailed schematic of dysregulated stem cell signaling pathways in triple-negative breast cancer (TNBC) and their link to immune evasion. The figure illustrates four key pathways—Notch, Wnt, Sonic Hedgehog (Shh), and TGF-β—converging on a central hub of pluripotency transcription factors (OCT4, SOX2, and NANOG) to drive self-renewal and tumorigenesis. Notch pathway: binding of the delta/jagged ligand to the NOTCH receptor triggers the release of the Notch Intracellular Domain (NICD). NICD translocates to the nucleus to maintain stemness and downregulate MHC Class I expression. This pathway can be targeted by the inhibitor MK0752. Wnt pathway: Wnt ligands bind to Frizzled/LRP receptors, leading to the intracellular accumulation and nuclear translocation of β-catenin. This signaling maintains stemness and actively modulates the tumor microenvironment by recruiting MDSCs and Tregs, while inhibiting dendritic cell (DC) maturation. Key inhibitors include OMP-54F28, LGK974, and Vantictumab. Sonic Hedgehog (Shh) Pathway: Shh binding to the Patched/Smoothened receptor complex activates the Gli transcription factor. Gli translocation maintains stemness and induces the production of the immunomodulatory cytokine IL-24, culminating in a diminished T cell response, immune system evasion, and tumor survival. Inhibitors targeting this pathway include Vismodegib and Sonidegib. TGF-β Pathway: The activated TGF-β ligand (released from the latent complex) binds to its receptors (TβR I and TβR II), leading to the phosphorylation of Smad2. The Smad2/Smad4 complex translocates to the nucleus to act as a coactivator, driving the production of further TGF-β. This cascade actively blocks Th1, Th2, and NK cell responses while increasing Treg activation. Conversely, TGF-β blockade is shown to reduce pluripotency.

Several key signaling pathways that govern normal stem cell differentiation and maintenance are frequently dysregulated in TNBC, contributing to its malignant characteristics and ability to evade the immune system. These include the Notch, Wnt/β-catenin, Sonic Hedgehog (Shh), and TGF-β/SMAD pathways (Table 1; Figure 2).

**TABLE 1:** Key stem cell signaling pathways and their dysregulation in TNBC immune response.

Signaling pathway	Normal function in stem cells	Signaling systems implicated	Dysregulation in TNBC	Impact on immune response in TNBC
Notch	Establishes cell fate, promotes cell division, aids in cell specialization, and ensures cell survival (Kontomanolis et al., 2018)	Interacts with the EGFR cascade and the Wnt/β-catenin system (Takebe et al., 2011; Li et al., 2017)	Upregulated; initiates EMT, promoting tumor invasion and dissemination (Li et al., 2017)	Associated with aggressive phenotypes and drug resistance (Pardo et al., 2024). Enhances cancer cell survival by preventing apoptosis and strengthening DNA repair mechanisms (McAuliffe et al., 2012)
Wnt/β-catenin	Regulates physiological processes, including cell proliferation, differentiation, and stem cell maintenance (Mir et al., 2025)	Crosstalk with the PI3K/AKT pathway, which can increase β-catenin’s nuclear translocation or block GSK-3β activity (Zhang and Wang, 2020)	Aberrant activation is linked to enhanced cancer stem cell characteristics, reduced apoptosis, higher cell proliferation, and treatment resistance (Merikhian et al., 2021)	Promotes an immunosuppressive TME, recruits MDSCs and Tregs, inhibits DC maturation, and increases PD-L1 expression (Patel et al., 2019)
Sonic Hedgehog (Shh)	Regulates biological processes in tissue stability and embryonic development, such as differentiation and proliferation (Mir et al., 2025)	Interacts with the Notch and Wnt/β-catenin pathways (Bhateja et al., 2019; Riobo-Del Galdo et al., 2019)	Aberrant activation promotes cancer stemness, cell proliferation, survival, angiogenesis, and metastasis; heavily stimulates CSCs (Bhateja et al., 2019; Riobo-Del Galdo et al., 2019)	Enhances the proliferation and invasion of TNBC cells; associated with poor prognosis and plays a potential role in immune evasion (Habib and O'Shaughnessy, 2016)
TGF-β/SMAD	Mediates growth inhibition, cell migration, invasion, EMT, extracellular matrix (ECM) remodeling, and immunological tolerance (Chen et al., 2021)	Intersects with Akt-PI3K, Wnt, Shh, Notch, interferon (IFN), and Ras signaling networks (Akhurst and Hata, 2012)	Acts as a tumor suppressor early in cancer development by inducing apoptosis, but later promotes stemness, proliferation, and invasiveness (Chen et al., 2021)	Decreases APC antigen exposure, suppresses Th1, Th2, and NK cells, and increases Treg activation (Quatromoni et al., 2013). Drives ECM development, physically blocking the infiltration of T cells, NK cells, and neutrophils into tumor tissues (Gorelik and Flavell, 2002; Wan and Flavell, 2007)

EGFR, epidermal growth factor receptor; EMT, epithelial-mesenchymal transition; TME, Tumor Microenvironment; MDSCs, Myeloid-derived suppressor cells; DC, dendritic cells; CSCs, Cancer Stem Cells; NK, natural killer; ECM, extracellular matrix.

The Notch signaling pathway plays a pivotal role in cell differentiation and the renewal of stem cells during development and tissue homeostasis. It is involved in regulating fundamental cellular processes such as cellular identity, proliferation, differentiation, and apoptosis (Pajcini et al., 2011). In the context of breast cancer, the dysregulation of Notch signaling components—including Notch receptors, their ligands, and their interactions—is implicated in tumor initiation, maintenance, and progression (Pardo et al., 2024). Aberrant Notch signaling in breast cancer is associated with abnormal tumorigenesis, modulation of vascular integrity, development of drug resistance, enhanced invasion, and increased migration of cancer cells (Ahmadi et al., 2024). Notably, high levels of Notch ligands and receptors have been correlated with poor prognosis in breast cancer patients, and the upregulation of Notch1 is strongly linked to the severe clinicopathological features observed in TNBC (Hossain et al., 2018).

Furthermore, Notch receptors are associated with the regulation of tumor-initiating cells (TICs)—a population considered synonymous with CSCs—by improving the cells’ capacity for self-renewal and promoting their growth, which is deeply implicated in the etiology of TNBC (Giuli et al., 2019; Takebe et al., 2011). By regulating genes associated with stem cell properties, including Sox2, Nanog, and Oct4, the Notch pathway contributes directly to the maintenance of CSCs in TNBC (Takebe et al., 2011). Consequently, the expression and activation of Notch receptors show a strong correlation with aggressive clinicopathological and biological phenotypes of breast cancer, such as invasiveness and chemoresistance, which are hallmark characteristics of the TNBC subtype (Hossain et al., 2018). Given the significant role of Notch signaling in TNBC, modulating this pathway represents a promising therapeutic strategy for this challenging disease (Pardo et al., 2024).

Parallel to Notch, the Wnt/β-catenin signaling pathway is another crucial regulator of stem cell self-renewal and differentiation in both adult tissues and during embryonic development (Bhavanasi and Klein, 2016). In TNBC, aberrant Wnt/β-catenin signaling is frequently observed and contributes to a myriad of malignant properties, including increased tumorigenicity, enhanced metastasis, and the development of therapeutic resistance (Serrano Garcia et al., 2024). Notably, the Wnt/β-catenin signaling pathway is more highly activated in TNBC compared to other breast cancer subtypes, and the accumulation of β-catenin in the nucleus of TNBC cells *in vitro* shows a strong positive correlation with tumor aggressiveness (King et al., 2012; Mahanujam, 2025). Dysfunction of the Wnt/β-catenin pathway at the cell surface can lead to its aberrant activation, which is thought to be a major driver of breast tumorigenesis (King et al., 2012). Furthermore, Wnt signaling in TNBC has been implicated in the process of metastasis, particularly to organs such as the lungs and brain (Hu et al., 2022). Importantly, Wnt signaling within CSCs in TNBC can also lead to an increase in the expression of PD-L1, a key immune checkpoint molecule, thereby contributing to the suppression of the immune response (Zheng et al., 2023). PD-L1 directly contributes to maintaining the stemness of CSCs by boosting BMI1 expression in a PI3K/AKT-independent manner and controlling Oct4A and Nanog expression in a PI3K/AKT-dependent manner (Almozyan et al., 2017).

Beyond driving stemness, the Wnt/β-catenin signaling pathway—primarily through the FZD receptor—significantly modulates the tumor microenvironment (TME) in TNBC, profoundly influencing the immune response through two primary mechanisms: promoting the recruitment of immunosuppressive cells and inhibiting the maturation of dendritic cells. In the first mechanism, the Wnt/β-catenin pathway modifies the TME to attract and foster immune-suppressive populations, such as myeloid-derived suppressor cells (MDSCs) and regulatory T cells (Tregs) (Katsuta et al., 2019). Secreted Wnt ligands facilitate cancer cell evasion from the immune system (Patel et al., 2019). High levels of β-catenin are associated with a non-inflamed phenotype, preventing T-cell infiltration (Li et al., 2019) and leading to the accumulation of immune-suppressive tumor-associated macrophages (TAMs) and T-cell exclusion. Moreover, MDSCs can differentiate into TAMs within the TME (Patel et al., 2019; Cha and Koo, 2020). Furthermore, epithelial-mesenchymal transition transcription factors (EMT-TFs), which are influenced by Wnt signaling, initiate the production of signaling molecules that attract Tregs, TAMs, and MDSCs (Taki et al., 2021). Overall, Wnt signaling promotes a state of immune tolerance (Patel et al., 2019). In TNBC cancer stem cells, Wnt signaling also increases the expression of PD-L1, further contributing to the immunosuppressive environment (Katoh and Katoh, 2022).

The second mechanism involves the pathway’s role in inhibiting the maturation of dendritic cells (DCs), which are critical antigen-presenting cells essential for T cell activation (Mediratta et al., 2020). Wnt ligands released by cancer cells activate the canonical Wnt signaling pathway in DCs, leading to changes in DC function that suppress immunity (Li et al., 2019). Specifically, the activation of β-catenin stimulates the transcriptional repressor ATF3, which inhibits CCL4 transcription. The resulting absence of CCL4 impairs the activation of CD103+ DCs, disrupting the subsequent activation and infiltration of crucial CD8+ T cells (Zheng et al., 2023). Wnt signaling in DCs also promotes the increased secretion of the immunosuppressive cytokine IL-10, restricts the production of the pro-inflammatory cytokine IL-12, and upregulates indoleamine 2,3-dioxygenase 1 (IDO1). These changes contribute to the production of Treg cells and inhibit cytotoxic T lymphocyte (CTL) activity (Li et al., 2019). Interestingly, the neutralization of Wnt has been shown to enhance T-cell responses, potentially by rescuing the activities of dendritic cells (Pacella et al., 2018).

Like Wnt and Notch, the Sonic Hedgehog (Shh) signaling pathway is essential for normal development and tissue homeostasis, playing a critical role in guiding cell differentiation, proliferation, and survival. However, aberrant activation of this pathway has been observed in various types of cancer, and this dysregulation is often linked to its role in regulating CSCs (Romaniuk-Drapala et al., 2024; Bhateja et al., 2019). Preclinical data are beginning to indicate that Shh may be essential for the preservation of the cancer stem cell phenotype, the activation of cancer-associated fibroblasts, invasive behavior, and angiogenesis in TNBC. The mechanism of activation is primarily non-canonical and involves the direct transcriptional upregulation of GLI1 and GLI2 (Bhateja et al., 2019).

Activation of the Shh pathway in TNBC cells has been shown to enhance their proliferation, invasion, and migration, while conversely, inhibiting Shh signaling can reduce the clonogenicity, self-renewal capacity, and motility of these cells. Notably, increased expression of GLI1, a key transcription factor downstream of Shh signaling, has been found to correlate with higher tumor stage and the presence of lymph node metastasis in TNBC patients (Habib and O'Shaughnessy, 2016). Strong evidence suggests that TNBC tumors are linked to Shh overexpression. These results were corroborated by immunostaining analysis, which revealed that increased expression of Shh in the TNBC subtype is associated with poor prognostic pathological features and a significantly reduced overall survival of patients (Noman et al., 2016). The co-activation of both the Shh and Wnt signaling pathways has also been identified as a poor prognostic marker in TNBC (Bhateja et al., 2019). Intriguingly, the Shh pathway appears to be involved in regulating the immune response, potentially playing a role in the ability of tumors to evade the host’s immune system (Palla et al., 2022).

Finally, the TGF-β/SMAD pathway controls several vital physiological processes, including immunological tolerance, tissue regeneration, homeostasis, development, cell migration, growth inhibition, and extracellular matrix (ECM) remodeling, all of which depend on TGF-β signaling (Chen et al., 2021). However, TGF-β also contributes to the development, growth, and metastasis of tumors. Overexpression of TGF-β can decrease the effectiveness of chemotherapy in advanced solid cancers. As a result, TGF-β signaling is crucial in producing an abnormal TME, which restricts the delivery of chemotherapeutic drugs and the effectiveness of anticancer therapies. Moreover, TGF-β secretion is encouraged by unequal drug distribution in the TME, which further reduces the effectiveness of treatment (Zhu et al., 2018).

In premalignant cells, TGF-β may function as a tumor suppressor. Through TGF-β/SMAD signaling and downstream effectors such as TGF-β-inducible early-response gene (TIEG1), SH2 domain-containing inositol-5-phosphatase, death-associated protein kinase 1 (DAPK1), and B-cell lymphoma 2 (BCL2), TGF-β induces apoptosis (Pardali and Moustakas, 2007). By causing cell cycle arrest through the control of cyclins, CDK inhibitors, and cyclin-dependent kinases (CDK), TGFβ signaling prevents the growth of cancer cells (Saltis, 1996; Hanahan, 2026). TGF-β-induced downregulation of c-Myc can cause cell cycle arrest in G1 and S phases by activating p21 and p15, in addition to the direct effects on cell cycle proteins (Ewen et al., 1995). These processes encourage apoptosis and prevent the growth of early cancer cells (Chen et al., 2021).

However, as malignancy progresses, pro-tumor immune cell types in the TME release cytokines like TGF-β and Tumor Necrosis Factor (TNF)-α that promote stemness and metastasis in TNBC. TGF-β, for its part, modulates apoptotic signals (Siegel and Massague, 2003) and controls CSCs (Phi et al., 2018) to promote tumor growth. Elevated levels of the anti-apoptotic protein Bcl-2 are seen in epirubicin-resistant TNBC cells, which lends credence to the theory that resistance entails apoptosis evasion. Because it can interact with TGF-β signaling and promote survival, the PI3K/Akt pathway further complicates this situation. Particularly, Akt has the ability to bind and sequester Smad3, preventing pro-apoptotic signals caused by TGF-β and reorienting the balance in favor of survival (Aashaq et al., 2022). In resistant TNBC cells, TGF-β and PI3K/Akt signaling work together to promote stemness and survival over cell death, as demonstrated by this dynamic interaction (Rosado-Sanz et al., 2025).

Furthermore, TGF-β suppresses the immune response through several distinct mechanisms: Antigen-presenting cells (APCs) are less exposed to antigens, and their activity is directly inhibited by TGF-β. While suppressing Th1, Th2, and NK cells, TGF-β also increases Treg activation. By preserving naïve T cells, TGF-β is crucial for T cell homeostasis. Additionally, TGF-β hinders the immune response by encouraging the development of extracellular matrix (ECM) and blocking the infiltration of immune cells, such as T cells, NK cells, and neutrophils, into tumor tissues (Chen et al., 2021).

While the dysregulation of these extracellular signaling networks—Notch, Wnt/β-catenin, Shh, and TGF-β/SMAD—provides the crucial external stimuli for maintaining the cancer stem cell niche and suppressing the immune microenvironment, these pathways do not act in isolation. Instead, they converge intracellularly to activate and sustain a core transcriptional circuitry. The ultimate effectors of these diverse signaling cascades are the embryonic transcription factors responsible for cellular plasticity. To fully understand how TNBC cells lock themselves into a malignant, immune-evasive, and self-renewing state, it is necessary to examine the downstream targets of these pathways. This directly underscores the critical role of key pluripotency regulators in orchestrating the aggressive phenotype of TNBC.

## 4 The role of key pluripotency regulators in TNBC

Several key pluripotency regulatory transcription factors, including Oct4, Sox2, Nanog, Klf4, and c-Myc, exhibit aberrant expression and function in TNBC, significantly contributing to its stem-like characteristics and aggressive clinical behavior (Table 2).

**TABLE 2:** Pluripotency regulatory transcription factors and their roles in TNBC immune response.

Transcription factor	Expression in TNBC	Key functions in TNBC	Role in immune evasion
OCT4	Overexpressed	Promotes stemness, self-renewal, and drug resistance; potentially mediates radiotherapy resistance	Downregulates MHC Class I; induces IL-24 production, which suppresses irradiation-induced premature senescence (Zhang et al., 2020; Kim et al., 2018)
SOX2	Overexpressed	Promotes stemness, EMT, and metastasis; increases cellular proliferation and tumorsphere formation (Leis et al., 2012)	Downregulates MHC Class I
NANOG	Overexpressed	Promotes stemness, self-renewal, tumorigenesis, and metastasis (Zeng et al., 2023; Zhang et al., 2016)	Downregulates MHC Class I; actively drives broad immune evasion mechanisms (Wang et al., 2013)
KLF4	Variable	Acts as a context-dependent tumor suppressor or promoter; induces apoptosis and prevents ubiquitination to enhance metastasis (Zou et al., 2019; Okuda et al., 2013). Interacts with SOX2 and OCT4 to control the ratio of differentiation to self-renewal, significantly affecting tumor progression and treatment response (Ayob and Ramasamy, 2018)	Downregulates MHC Class I
MYC	Overexpressed	Promotes cell growth, stemness, drug resistance, and metabolic reprogramming to ensure survival in nutrient-poor conditions; overexpression elevates lipid and amino acid metabolism (Dong et al., 2020)	Downregulates MHC Class I via the suppression of B2M and NLRC5 (Zheng et al., 2023); represses STING-dependent innate immunity, actively driving a non-inflamed TIME (Wu et al., 2021)

MHC, major histocompatibility complex; EMT, epithelial-mesenchymal transition; B2M, Beta-2, Microglobulin; NLRC5, NOD-like receptor family CARD, domain containing 5; TIME, tumor immune microenvironment.

Oct4 is frequently overexpressed in TNBC, directly promoting the pluripotency and self-renewal of tumor cells (Fultang et al., 2021). Studies have linked Oct4 to breast cancer stem-like cells; specifically, CD44+/CD24− tumor populations are highly enriched with stem and progenitor characteristics. By evaluating these breast cancer stem cells, researchers have been able to distinguish between CD44+/CD24− and non-CD44+/CD24− tumor cells based on the expression of seven specific genes: CD44, Oct4, nestin, APC, CDC2, HGF, and TGF-β (Liu et al., 2011). This implies that Oct4 expression is intimately connected to carcinogenesis and self-renewal through the activation of its downstream target genes, making it a potential biomarker for the development, dissemination, and differentiation of breast cancer (Liu et al., 2011). Furthermore, Oct4 expression serves as an independent poor prognostic factor. In hormone receptor-positive breast cancers, Oct4 is substantially correlated with ALDH1 expression, tamoxifen resistance, aggressive tumor characteristics, and poor clinical prognosis (Gwak et al., 2017). Therapeutically, targeting this factor shows promise; for instance, the bioactive compound bufalin has been demonstrated to considerably reduce the stemness of TNBC cells by inhibiting the expression of both Sox2 and Oct4, concurrently preventing their proliferation and inducing apoptosis (Chen et al., 2020). Interestingly, Oct4 is upregulated in normal breast tissue during lactation, and its expression further increases in breast tumors exhibiting lactating features—a subtype that often overlaps with triple-negative cancers. Oct4 co-localizes with Nanog in breast cancers and confers a tumorigenic nature when expressed at levels above the normal physiological range (Hassiotou et al., 2012; Hassiotou et al., 2013). Oct4 is also a critical component of the Stat3/Oct4/c-Myc signaling pathway that controls stemness. In doxorubicin (Dox)-resistant TNBC cells, both Oct4 and c-Myc are highly expressed alongside increased Stat3 phosphorylation, which further promotes the enrichment of CSCs (Cheng et al., 2018). The vital role of Oct4 in BCSC survival is underscored by findings from Sen et al., which demonstrated that siRNA-mediated Oct4 knockdown *in vitro* alters BCSC morphology, prevents mammosphere formation, and induces cell death (Sen et al., 2023). Additionally, Oct4 is involved in the complex regulation of CSCs and can be upregulated by the Shh signaling pathway. Beyond driving stemness, Oct4 can induce the production of IL-24 through the activation of STAT3 and NF-κB signaling pathways, ultimately conferring radiotherapy resistance in breast cancer cells (Zhang et al., 2020; Kim et al., 2018).

Parallel to Oct4, Sox2 is heavily overexpressed in TNBC, further contributing to the pluripotency and self-renewal of tumor cells (Fultang et al., 2021). Its expression is commonly associated with high histologic grade, a high Ki-67 proliferation index, and p53 overexpression in breast cancer (Gwak et al., 2017). As previously noted, agents like bufalin can inhibit Sox2 expression to effectively reduce TNBC cell stemness (Chen et al., 2020). Notably, Sox2 expression in TNBC is closely correlated with the epithelial-mesenchymal transition (EMT), the induction of CSC features, and resistance to gamma-secretase inhibitors (GSIs) (Fournier et al., 2024). The observation that Sox2 is strongly expressed specifically in breast cancer tissues and lymph nodes, where the tumor has spread, suggests it plays a critical role in metastatic dissemination (Li et al., 2013). To facilitate this tumor spread via EMT, Sox2 preferentially leverages Wnt/β-catenin signals, rather than the TGF-β and Snail signaling pathways. In both human breast and prostate cancers, Sox2 directly regulates the transcription of β-catenin by binding to its promoter region (Li et al., 2013). Clinically, high levels of Sox2 expression in TNBC patients are linked to shorter overall and disease-free survival. Conversely, inhibiting Sox2 suppresses cell proliferation and invasion, induces apoptosis in TNBC cells *in vitro*, and reduces tumorigenesis and metastasis *in vivo* (Liu et al., 2018). In human triple-negative breast tumors, SOX2 and TWIST1 serve as master regulators of CSC characteristics. Specifically, siRNA knockdown studies and chemosensitivity tests have revealed that the SOX2-ABCG2-TWIST1 axis is crucial for controlling chemoresistance and carcinogenicity in TNBC stem cells (Mukherjee et al., 2017). This SOX2 overexpression in TNBC stem cells may be driven by an IMP3/SLUG signaling axis that specifically transcribes the gene (Samanta et al., 2016). Beyond promoting stemness, high expression of Sox2 in breast cancer cells stimulates nuclear factors of activated T cells (NFAT), STAT3, and NF-kB signaling to release chemokines (such as CCL3) and intercellular adhesion molecule-1 (ICAM-1). These molecules actively attract tumor-associated macrophages (TAMs) into the tumor microenvironment, further facilitating tumor metastasis (Mou et al., 2015; Chaturvedi et al., 2014).

Working closely alongside Oct4 and Sox2, Nanog is another core pluripotency regulator overexpressed in TNBC, heavily promoting the stemness and self-renewal of tumor cells (Fultang et al., 2021). Higher Nanog expression in TNBC patients is directly associated with a poorer prognosis, highlighting its value as a prognostic biomarker (Nagata et al., 2017). Research demonstrates that in breast cancer, Nanog functions as a transcription factor controlling gene networks that encourage the growth and metastasis of mammary tumors (Lu et al., 2014). Mechanistically, a protein complex consisting of Cx26, Nanog, and FAK has been identified in TNBC patient samples and is believed to be essential for tumorsphere formation, CSC maintenance, and Nanog protein stability. Disrupting this complex with a cell-penetrating peptide derived from Cx26 (aCx26-pep) reduces nuclear FAK and Nanog levels, inhibiting the expression of Nanog target genes and severely disrupting self-renewal in TNBC cells (Mulkearns-Hubert et al., 2024). Furthermore, within TNBC cell lines, cells expressing high levels of a Nanog-driven GFP reporter (Nanog-GFP+ cells) concurrently exhibit elevated levels of Oct4 and Sox2, along with enhanced self-renewal and tumor initiation capacities. These highly aggressive cells also express mesenchymal markers and show increased invasiveness (Thiagarajan et al., 2015). Ultimately, Nanog is a master regulator governing several aspects of cancer progression, including tumor cell proliferation, self-renewal, motility, EMT, immune evasion, and drug resistance (Wang et al., 2013).

While Oct4, Sox2, and Nanog uniformly promote stemness, the role of Klf4 in TNBC presents a more complex and context-dependent paradigm, acting as both a tumor suppressor and a promoter (Roberts et al., 2020). For example, Klf4 acts as a transcriptional activator of NOXA; applying the Klf4 activator APTO-253 causes apoptosis in TNBC cells with p53 mutations by upregulating NOXA expression (Ferralli et al., 2016). Similarly, one study indicated that higher Klf4 expression in TNBC patients was associated with a better prognosis due to the suppression of EMT (Nagata et al., 2016). Klf4 negatively regulates the migratory, invasive, and proliferative characteristics of metastases in part by suppressing the expression of the EGFR gene (Roberts et al., 2020), and its expression is often significantly decreased in breast infiltrating ductal carcinoma tissues and cell lines (Wang et al., 2015). In contrast, nuclear localization of Klf4 has been linked to an aggressive phenotype in early-stage breast cancer (Pandya et al., 2004). This duality is partially explained by an alternative splice variant, Klf4α, which acts as an antagonist to full-length Klf4 (FL). By sequestering Klf4 (FL) in the cytoplasm, Klf4α inhibits its nuclear tumor-suppressive properties (such as growth inhibition) and stimulates breast cancer cell proliferation (Roberts et al., 2020). Furthermore, Klf4 interacts dynamically with epigenetic modifiers. Normal breast cells epigenetically suppress Vascular Endothelial Growth Factor (VEGF) transcription through the sequential binding of KLF-4 and HDACs to the VEGF promoter (Ray et al., 2013). However, Guo et al. demonstrated that in breast cancer, the de-repression of this KLF-4-HDAC molecular switch not only lifts the transcriptional suppression of VEGF but also actively allows for a SAF-1-mediated transcriptional surge of VEGF, driving critical tumor angiogenesis (Guo et al., 2018).

Finally, unlike the dual nature of Klf4, the transcription factor c-Myc acts as a definitive oncogenic driver and is frequently elevated in TNBC compared to other subtypes. Higher levels of c-Myc protein and its phosphorylated form are observed in TNBC tumors compared to receptor-positive tumors, making CDK inhibitors a highly rational therapeutic option against aggressive c-Myc-overexpressing triple-negative tumors (as well as luminal B cancers) (Horiuchi et al., 2012). Overexpression of c-Myc is strongly associated with poorer outcomes and actively contributes to the development of drug resistance in TNBC (Klauber-DeMore et al., 2018). Notably, inhibiting c-Myc directly depletes CSCs in TNBC by inducing cellular senescence (Yang et al., 2017). In Dox-resistant TNBC cells, increased phosphorylation of Stat3 drives the high expression of the pluripotent transcription factors Oct4 and c-Myc, facilitating the robust enrichment of CSCs (Cheng et al., 2018). Given that c-Myc is dysregulated in a large proportion of cancers and is the most frequently amplified gene in breast cancer (Tang et al., 2022), its high mRNA expression—even independent of DNA amplification—is significantly associated with worse overall survival in TNBC patients (Katsuta et al., 2019). Importantly, c-Myc also actively shapes the tumor-immune landscape. Amplification and overexpression of c-Myc show a strong correlation with a non-inflamed tumor microenvironment and low immune infiltration. Wu et al. were the first to demonstrate that c-Myc directly suppresses STING-dependent innate immunity in TNBC cells, actively causing immunological escape. Because c-Myc possesses the highest incidence of amplification in TNBC, researchers postulate that its negative regulation of STING is a primary driver of the non-inflammatory, immune-excluded microenvironment characteristic of this disease (Wu et al., 2021).

Collectively, the aberrant expression of these master transcription factors—Oct4, Sox2, Nanog, Klf4, and c-Myc—locks TNBC cells into a highly plastic, chemoresistant, and metastatic state. However, the influence of these pluripotency regulators extends far beyond maintaining the cancer stem cell niche. As briefly observed with Sox2, Nanog, and c-Myc, these factors actively sculpt the tumor microenvironment, suppressing host immunity to protect vulnerable CSC populations from clearance. Understanding this crosstalk is critical. The following chapter will explore the specific mechanisms by which these pluripotency regulators orchestrate immune evasion in TNBC, effectively shielding the tumor from immune-mediated destruction.

## 5 Mechanisms of immune evasion by pluripotency regulators in TNBC

Pluripotency regulatory transcription factors (Oct4, Sox2, Nanog, Klf4, and c-Myc) enable TNBC cells to evade the host’s immune system primarily through two mechanisms: downregulating antigen presentation molecules, specifically the major histocompatibility complex (MHC) class I, and producing immunosuppressive cytokines. MHC class I molecules are crucial for presenting tumor-associated antigens to cytotoxic T lymphocytes (CTLs), the primary effectors of anti-tumor immunity. By reducing the surface expression of MHC class I, TNBC cells effectively become less visible to CTLs, hindering their recognition and subsequent immune-mediated killing (Table 2). A specific, well-established mechanism of impaired antigen presentation involves c-Myc, which is frequently overexpressed in TNBC and negatively correlates with the expression of important MHC-I presentation genes, such as B2M and NLRC5 (Zheng et al., 2023; Lee et al., 2022). However, the specific, direct contributions of other pluripotency regulators like Oct4, Sox2, Nanog, and Klf4 to MHC class I downregulation require further elucidation beyond the currently available evidence.

In addition to reducing antigen presentation, these pluripotency regulators contribute to immune evasion by promoting the production of immunosuppressive cytokines. These cytokines act locally within the tumor microenvironment (TME) to dampen the activity of anti-tumor immune cells, such as CTLs and natural killer (NK) cells, while simultaneously promoting the recruitment and activity of immunosuppressive populations like myeloid-derived suppressor cells (MDSCs) and regulatory T cells (Tregs).

For instance, Notch and USP9x have been shown to work together to drive the TME stress response, inflammation, and immune evasion in TNBC, resulting in the secretion of proinflammatory cytokines and the recruitment of pro-tumoral macrophages (Jaiswal et al., 2021). Furthermore, Nanog actively regulates immune evasion (Wang et al., 2013), with the Nanog-dependent Tcl1a/Akt pathway mediating immunological resistance and enhancing the stem-like phenotype in tumor cells (Noh et al., 2012). However, the specific immunosuppressive cytokines that Nanog might induce in TNBC are not fully detailed in the current literature. Similarly, Oct4’s function is strongly linked to the STAT3 signaling pathway, with its expression correlating positively with STAT3 activity in various cancer stem cells (Cheng et al., 2018; Kim et al., 2013). Interleukin-6 (IL-6) activates the JAK1/STAT3 pathway, which in turn drives Oct4 expression to promote breast CSCs. Oct4 also stimulates the STAT3 and NF-κB pathways to produce IL-24 (Kim et al., 2018). Although IL-24 possesses diverse anticancer properties—such as the inhibition of tumor cell invasion and metastasis, anti-angiogenic action, immunological modulatory activity, “bystander” antitumor activity, and the specific inhibition of tumor cell growth while inducing apoptosis (Zheng et al., 2007; Deng et al., 2020) —this cytokine paradoxically confers resistance to radiation therapy by preventing premature senescence (Kim et al., 2018). Further investigation is required to fully characterize the specific immunosuppressive cytokines produced by Oct4, Sox2, Klf4, and c-Myc in TNBC and their precise roles in mediating immune evasion.

Given that these pluripotency networks not only drive the intrinsic malignancy of TNBC but also actively orchestrate its escape from immune surveillance, they represent highly attractive vulnerabilities. Disrupting these master regulators could simultaneously strip the tumor of its stem-like plasticity and unmask it to the host immune system. Consequently, evaluating how these molecular insights can be translated into clinical interventions is critical, as explored in the following section on potential immunotherapeutic strategies targeting pluripotency regulators and their associated pathways in TNBC.

## 6 Potential immunotherapeutic strategies targeting pluripotency regulators and associated pathways in TNBC

Given the significant roles of pluripotency regulators and their associated signaling pathways in driving TNBC progression and mediating immune evasion, targeting these molecules and pathways represents a promising avenue for the development of novel immunotherapeutic strategies (Table 3; Figure 2).

**TABLE 3:** Potential immunotherapeutic strategies targeting pluripotency regulators and associated pathways in TNBC.

Therapeutic strategy	Description	Relevant clinical/preclinical findings
Direct targeting of Oct4, Sox2, Nanog, and Myc	Utilizing specific inhibitors, peptide-based approaches, or targeting upstream regulators like STAT3 or MCL1 (Zhang et al., 2020)	The peptide aCx26-pep targeting the NANOG/FAK interaction shows preclinical promise (Mulkearns-Hubert et al., 2024); the STAT3 inhibitor WP1066 reduces Oct4/c-MYC in doxorubicin-resistant TNBC cells (Cheng et al., 2018); MCL1 inhibition downregulates Oct4/Sox2 (Pratelli et al., 2023)
Modulation of Notch signaling	Utilizing GSIs or directly targeting specific Notch receptors and ligands (Pardo et al., 2024)	AL101 monotherapy is currently being evaluated in clinical trials for Notch-activated recurrent or metastatic TNBC (e.g., NCT04461600)
Inhibition of Wnt/β-catenin signaling	Utilizing small molecule inhibitors targeting various pathway components (e.g., Wnt ligands, Frizzled receptors, or intracellular β-catenin) (King et al., 2012)	Wnt inhibitors demonstrate significant promise in preclinical studies (Luke et al., 2019); and show the potential to enhance tumor susceptibility to concurrent immunotherapy (King et al., 2012)
Targeting Sonic Hedgehog (Shh) signaling	Utilizing inhibitors directed against SMO, GLI transcription factors, or the Shh ligand itself (Bhateja et al., 2019)	Preclinical studies indicate that inhibition reduces TNBC cell proliferation and invasion (Habib and O'Shaughnessy, 2016); clinical trials evaluating SMO inhibitors in TNBC are currently underway (Bhateja et al., 2019)
Inhibition of TGF-β signaling	Utilizing anti-fibrotic agents or specific pathway inhibitors to suppress TGF-β expression and block downstream SMAD phosphorylation	Pirfenidone (PFD) can restore the extracellular matrix and enhance the efficacy of chemotherapy. PFD inhibits fibroblast growth by decreasing pSmad2/3 and downregulating TGF-β expression (Ji et al., 2013)

STAT3, Signal Transducer and Activator of Transcription 3; MCL1, Myeloid Cell Leukemia-1; GSIs, gamma-secretase Inhibitors; SMO, smoothened; SMAD, suppressor of mothers against decapentaplegic.

Directly targeting Oct4, Sox2, and Nanog transcription factors is currently being explored as a potential therapeutic approach in various cancers, including TNBC (Zhang et al., 2020). For instance, a peptide-based strategy that specifically targets the interaction between Nanog and FAK within TNBC cells has shown promising therapeutic potential in preclinical models (Mulkearns-Hubert et al., 2024). Additionally, WP1066, an inhibitor of the STAT3 signaling pathway, has demonstrated the ability to overcome doxorubicin resistance in TNBC by directly reducing the expression of both Oct4 and c-Myc (Cheng et al., 2018). Furthermore, inhibiting MCL1, an antiapoptotic protein, in TNBC cells has been shown to downregulate the expression of Oct4 and Sox2, leading to a substantial suppression of tumor invasiveness (Djamgoz, 2025). These findings suggest that both direct and indirect targeting of pluripotency regulators can exert potent anti-tumor effects in TNBC.

Modulating the activity of key upstream stem cell signaling pathways—such as Notch, Wnt/β-catenin, and Shh—also holds significant promise for enhancing anti-tumor immunity. Given the upregulation and protumorigenic roles of the Notch signaling pathway in TNBC, its modulation is frequently attempted using Gamma-Secretase Inhibitors (GSIs). Molecules such as MK-0752 and RO4929097 are designed to bind directly to the gamma-secretase enzyme and disable it, physically blocking the release of the Notch Intracellular Domain (NICD) so that it never reaches the nucleus to activate Nanog or Sox2 (Pardo et al., 2024). MK-0752 (developed by Merck) was one of the first GSIs to demonstrate the possibility of specifically targeting cancer stem cells in humans (when used in combination with docetaxel); however, it presented a high toxicity profile, causing severe gastrointestinal side effects (Schott et al., 2013). In contrast, Roche developed RO4929097 as a more potent, selective, and “cleaner” version of the drug, often evaluated in combination with paclitaxel and carboplatin as a neoadjuvant therapy (Sardesai et al., 2020). Despite early promise, its development was discontinued due to its intrinsic interference with gut cell renovation (specifically goblet cells) required for mucus production, which caused severe diarrhea.

Similarly, the Wnt/β-catenin pathway, which is frequently overactivated in TNBC and contributes heavily to immune suppression, is being actively investigated as a therapeutic target (King et al., 2012). Inhibition of this pathway has shown potential in boosting the overall immune response and could potentially make immunologically “cold” tumors, which are typically unresponsive to standard immunotherapy, much more susceptible to treatment (Rogaczewski et al., 2022). The Sonic Hedgehog (Shh) signaling pathway, known for its role in regulating CSCs, is also being explored as a therapeutic target in various cancers, including TNBC, to inhibit CSC activity and halt overall tumorigenesis (Bhateja et al., 2019).

Furthermore, the tumor microenvironment itself presents therapeutic targets, particularly regarding TGF-β. When TNBC is treated with the chemotherapeutic drug paclitaxel, autocrine TGF-β signaling is hyperactivated, which paradoxically increases therapy resistance and the likelihood of relapse (Bhola et al., 2013). Infiltrating stromal and immune cell populations can also produce TGF-β, rendering it frequently abundant in the TNBC microenvironment (Bierie et al., 2008). Current findings strongly point to CSC accumulation driven by TGF-β as a primary mechanism of treatment resistance in TNBC (Fultang et al., 2021). Addressing this, a novel anti-fibrotic drug called pirfenidone (PFD) can improve the effectiveness of chemotherapy while rebuilding the extracellular matrix. PFD suppresses fibroblast proliferation by decreasing pSmad2/3 and downregulating TGF-β expression (Ji et al., 2013). Notably, PFD therapy administered in conjunction with doxorubicin successfully reduced cancer-associated fibroblasts (CAFs) and slowed tumor growth in a TNBC model (Takai et al., 2016). While pharmacological inhibitors targeting these pluripotency pathways demonstrate clear mechanistic rationale, their clinical translation is frequently hindered by the severe, dose-limiting systemic toxicities associated with disrupting vital developmental pathways in healthy tissues (as observed with GSIs). To achieve the precise eradication of therapy-resistant cancer stem cells without debilitating off-target effects, the field is increasingly turning toward highly specific, engineered cellular interventions. These imperative paves the way for advanced adoptive cell therapies, leading us to examine the potential use of CAR-T therapy for TNBC.

## 7 The potential use of CAR-T therapy for TNBC

In response to the limitations of conventional systemic treatments, chimeric antigen receptor T-cell (CAR-T) therapy has emerged as a highly promising therapeutic alternative for triple-negative breast cancer (TNBC). Recent research has validated the feasibility of designing CAR-T cells directed against antigens overexpressed in solid tumors, such as EGFR and HER2. As evidence of this, data from clinical trials conducted in 2023 demonstrated that HER2-targeted CAR-T therapy significantly reduced tumor burden in patients with metastatic breast cancer, yielding encouraging clinical response rates (He et al., 2023). Although bioengineering innovations have improved the therapeutic index of these therapies—enhancing *in vivo* cellular persistence and mitigating systemic toxicity—their efficacy in TNBC remains hindered by antigenic heterogeneity and the formidable immunosuppressive barriers imposed by the TME (Kausar et al., 2023).

To counteract this resistance, current clinical research has pivoted toward the development of combinatorial strategies, highlighting the profound biological synergy between CAR-T therapy and immune checkpoint inhibitors (ICIs). This combination operates through a complementary mechanism: CAR-T cells specifically target the tumor and promote immune infiltration, helping to inflame previously “cold” or poorly immunogenic lesions. Simultaneously, blockade of the PD-1/PD-L1 pathway alleviates the exhaustion of the CAR-T cells themselves and reverses the immunosuppressive conditions of the TME, thereby fostering much more durable anti-tumor activity. In addition to the traditional systemic co-administration of antibodies (Mahendran et al., 2024) cutting-edge advancements have led to fourth-generation “armored” CAR-T cells, which are genetically engineered to locally secrete anti-PD-1 single-chain variable fragments (scFv) exclusively upon activation at the tumor site. This inducible, intratumoral release not only enhances the effector function of both CAR-T cells and endogenous tumor-infiltrating lymphocytes (TILs) but also circumvents systemic PD-1 blockade, thereby minimizing the risk of autoimmune toxicity. Altogether, the integration of CAR-T specific targeting with the immunological restoration facilitated by PD-L1 blockade represents a fundamental pillar in the evolving therapeutic landscape of TNBC.

## 8 Current clinical research and recent findings

While advanced cellular therapies like CAR-T represent the cutting edge of precision oncology, their ultimate success relies on a broader understanding of the systemic immunotherapies and targeted agents currently being evaluated in patients. Expanding upon these targeted approaches, current clinical research is actively exploring the potential of immunotherapy for the treatment of TNBC, and several ongoing studies are shedding crucial light on the interplay between pluripotency regulators, their associated pathways, and the immune system.

Several active clinical trials are focusing on the use of immunotherapy, often in combination with chemotherapy, for patients with TNBC. These trials, many of which are supported by the National Cancer Institute (NCI), are investigating the efficacy and safety of various ICIs, such as pembrolizumab, alone or in combination with different chemotherapy regimens, in both early-stage and metastatic TNBC (Sriramulu et al., 2024). One notable study is evaluating the combination of sacituzumab govitecan, an antibody-drug conjugate, with pembrolizumab in patients with residual invasive disease after surgery and neoadjuvant therapy. Interestingly, a clinical trial is also underway to evaluate the use of AL101 as a monotherapy in patients with recurrent or metastatic TNBC whose tumors exhibit Notch pathway activation. This specific trial highlights the growing recognition of the clinical relevance of dysregulated stem cell signaling pathways, such as Notch, in this disease. Furthermore, the efficacy of olaparib, a PARP inhibitor, and atezolizumab, a PD-L1 inhibitor, is being investigated both as single agents and in combination for the treatment of stage III-IV TNBC. The combination of atezolizumab with chemotherapy has already demonstrated significant overall survival benefits in patients with advanced TNBC whose tumors express PD-L1 (Sriramulu et al., 2024), marking an important step forward in the management of this aggressive malignancy.

Recent research findings are further illuminating the complex interactions between various molecular factors and the immune system in TNBC. For instance, the expression of the chemokine receptor CCR5 has been associated with a better prognosis in TNBC and shows a positive correlation with the infiltration of immune cells into the tumor and the activity of tumor immune response-related pathways (Wang et al., 2021). Similarly, regulatory T cells (Tregs) are deeply associated with the tumor immune microenvironment and the overall response to immunotherapy in TNBC (Huang et al., 2023). Notably, the activation of the Wnt/β-catenin pathway has been correlated with a distinct increase in the presence of TAMs within the TME. Preclinical studies have also shown that sequential immunotherapy targeting both PD-L1 and IL-6R can effectively reduce TNBC aggressiveness and mortality, particularly when utilized in combination with MCT-1 inhibition (Haq et al., 2024). These recent findings underscore the dynamic and intricate nature of the immune landscape in TNBC and suggest potential biomarkers and therapeutic targets for improving immunotherapy outcomes.

Finally, navigating the modulation of these pathways in a clinical setting requires nuance, particularly regarding paradoxical signaling. According to a study by Zhu et al., administering TGF-βI inhibitors to TNBC patients inadvertently raised the expression of mesenchymal markers and decreased the expression of epithelial markers, suggesting greater invasion, migration, and metastatic potential. Conversely, a different study demonstrated that the anti-inflammatory drug ophiopogonin D interferes with the TGF-βI pathway, which ordinarily stimulates ITGB1/FAK/Src/AKT signaling. Ultimately, despite the complex dynamics of the TME, carefully deployed TGF-β inhibitors remain effective treatments for counteracting the pro-metastatic alterations induced by TGF-β signaling in TNBC patients (Zhu et al., 2020).

## 9 Conclusion and future perspectives

Triple-negative breast cancer remains a formidable clinical challenge characterized by its aggressive clinical course, high metastatic propensity, and a critical lack of conventionally targeted therapeutic options. A defining biological hallmark of TNBC is the profound enrichment of cancer stem cells (CSCs), which serve as the primary engines of tumor progression, acquire treatment resistance, and lead to early recurrence. The plasticity and pluripotency of these CSCs are intricately maintained by a core network of embryonic transcription factors—namely Oct4, Sox2, Nanog, Klf4, and c-Myc—that are frequently overexpressed in this malignancy. Crucially, the aberrant activation of these regulators extends far beyond promoting self-renewal; they actively engineer a shielded tumor microenvironment (TME) to facilitate immune evasion. By downregulating MHC class I antigen presentation molecules and driving the secretion of immunosuppressive cytokines, these transcription factors effectively render the tumor invisible to cytotoxic T lymphocytes. Furthermore, upstream signaling networks, particularly the frequently dysregulated Wnt/β-catenin pathway, critically modulate the TME by dampening the maturation of dendritic cells and recruiting immunosuppressive populations, such as MDSCs and Tregs, thereby deeply fracturing the anti-tumor immune response.

The growing mechanistic understanding of how these pluripotency regulators and their associated signaling axes operate in TNBC has unveiled a compelling new frontier for therapeutic intervention. Direct pharmacological targeting of these master transcription factors, alongside the modulation of key pathways like Notch, Wnt/β-catenin, and Shh, is actively being explored to strip TNBC of its stem-like defenses and sensitize it to host immunity. Current clinical research reflects this paradigm shift, rigorously investigating the efficacy of immune checkpoint inhibitors (ICIs)—often deployed synergistically with conventional chemotherapy—while cutting-edge cellular interventions, such as CAR-T therapy, are being refined to overcome the profound immunosuppressive barriers of the TME. Together, these recent findings continue to unravel the complex interplay between tumor plasticity and immune suppression, identifying vital biomarkers to improve patient outcomes.

Ultimately, future research must prioritize elucidating the precise, gene-specific mechanisms by which each key pluripotency regulator orchestrates immune evasion in TNBC. To achieve this, the integration of advanced omics sciences and high-resolution immunomonitoring will be critical for mapping these complex transcriptional networks and stratifying patients for personalized management. Furthermore, investigating rational combinatorial strategies—such as pairing direct pluripotency network inhibitors or advanced cellular therapies with existing immune checkpoint blockades—holds immense promise for preventing CSC-driven relapse and achieving durable, long-term responses in patients facing this highly aggressive disease.

## References

[B1] Aashaq S. Batool A. Mir S. A. Beigh M. A. Andrabi K. I. Shah Z. A. (2022). TGF-Beta signaling: a recap of SMAD-independent and SMAD-dependent pathways. J. Cell. Physiol. 237 (1), 59–85. 10.1002/jcp.30529 34286853

[B2] Ahmadi A. Moqadami A. Khalaj-Kondori M. Ghiasvand S. (2024). Non-coding RNAs affect breast cancer development through the notch signaling pathway: an overview. Gene Expr. 23 (1), 44–56. 10.14218/ge.2023.00084

[B3] Akbarzadeh M. Maroufi N. F. Tazehkand A. P. Akbarzadeh M. Bastani S. Safdari R. (2019). Current approaches in identification and isolation of cancer stem cells. J. Cell. Physiol. 234 (9), 14759–14772. 10.1002/jcp.28271 30741412

[B4] Akhurst R. J. Hata A. (2012). Targeting the TGFbeta signalling pathway in disease. Nat. Rev. Drug Discov. 11 (10), 790–811. 10.1038/nrd3810 23000686 PMC3520610

[B5] Al-Hajj M. Wicha M. S. Benito-Hernandez A. Morrison S. J. Clarke M. F. (2003). Prospective identification of tumorigenic breast cancer cells. Proc. Natl. Acad. Sci. U. S. A. 100 (7), 3983–3988. 10.1073/pnas.0530291100 12629218 PMC153034

[B6] Almozyan S. Colak D. Mansour F. Alaiya A. Al-Harazi O. Qattan A. (2017). PD-L1 promotes OCT4 and Nanog expression in breast cancer stem cells by sustaining PI3K/AKT pathway activation. Int. J. Cancer 141 (7), 1402–1412. 10.1002/ijc.30834 28614911 PMC5575465

[B7] Anders C. Carey L. A. (2008). Understanding and treating triple-negative breast cancer. Oncol. (Williston Park) 22 (11), 1233–1239. 18980022 PMC2868264

[B8] Ayob A. Z. Ramasamy T. S. (2018). Cancer stem cells as key drivers of tumour progression. J. Biomed. Sci. 25 (1), 20. 10.1186/s12929-018-0426-4 29506506 PMC5838954

[B9] Bhateja P. Cherian M. Majumder S. Ramaswamy B. (2019). The hedgehog signaling pathway: a viable target in breast cancer? Cancers (Basel) 11 (8), 1126. 10.3390/cancers11081126 31394751 PMC6721501

[B10] Bhavanasi D. Klein P. S. (2016). Wnt signaling in normal and malignant stem cells. Curr. Stem Cell. Rep. 2 (4), 379–387. 10.1007/s40778-016-0068-y 28503404 PMC5423672

[B11] Bhola N. E. Balko J. M. Dugger T. C. Kuba M. G. Sánchez V. Sanders M. (2013). TGF-beta inhibition enhances chemotherapy action against triple-negative breast cancer. J. Clin. Invest. 123 (3), 1348–1358. 10.1172/JCI65416 23391723 PMC3582135

[B12] Bianchini G. Balko J. M. Mayer I. A. Sanders M. E. Gianni L. (2016). Triple-negative breast cancer: challenges and opportunities of a heterogeneous disease. Nat. Rev. Clin. Oncol. 13 (11), 674–690. 10.1038/nrclinonc.2016.66 27184417 PMC5461122

[B13] Bierie B. Stover D. G. Abel T. W. Chytil A. Gorska A. E. Aakre M. (2008). Transforming growth factor-beta regulates mammary carcinoma cell survival and interaction with the adjacent microenvironment. Cancer Res. 68 (6), 1809–1819. 10.1158/0008-5472.CAN-07-5597 18339861

[B14] Cha Y. J. Koo J. S. (2020). Role of tumor-associated myeloid cells in breast cancer. Cells 9 (8), 1785. 10.3390/cells9081785 32726950 PMC7464644

[B15] Chaturvedi P. Gilkes D. M. Takano N. Semenza G. L. (2014). Hypoxia-inducible factor-dependent signaling between triple-negative breast cancer cells and mesenchymal stem cells promotes macrophage recruitment. Proc. Natl. Acad. Sci. U. S. A. 111 (20), E2120–E2129. 10.1073/pnas.1406655111 24799675 PMC4034192

[B16] Chen F. Zhu L. Hu J. Jiang S. Liu H. Zheng J. (2020). Bufalin attenuates triple-negative breast cancer cell stemness by inhibiting the expression of SOX2/OCT4. Oncol. Lett. 20 (5), 171. 10.3892/ol.2020.12028 32934738 PMC7471667

[B17] Chen J. Ding Z. Y. Li S. Liu S. Xiao C. Li Z. (2021). Targeting transforming growth factor-beta signaling for enhanced cancer chemotherapy. Theranostics 11 (3), 1345–1363. 10.7150/thno.51383 33391538 PMC7738904

[B18] Cheng C. C. Shi L. H. Wang X. J. Wang S. X. Wan X. Q. Liu S. R. (2018). Stat3/Oct-4/c-Myc signal circuit for regulating stemness-mediated doxorubicin resistance of triple-negative breast cancer cells and inhibitory effects of WP1066. Int. J. Oncol. 53 (1), 339–348. 10.3892/ijo.2018.4399 29750424

[B19] de Ruijter T. C. Veeck J. de Hoon J. P. J. van Engeland M. Tjan-Heijnen V. C. (2011). Characteristics of triple-negative breast cancer. J. Cancer Res. Clin. Oncol. 137 (2), 183–192. 10.1007/s00432-010-0957-x 21069385 PMC3018596

[B20] Deng L. Fan J. Ding Y. Yang X. Huang B. Hu Z. (2020). Target therapy with Vaccinia virus harboring IL-24 for human breast cancer. J. Cancer 11 (5), 1017–1026. 10.7150/jca.37590 31956348 PMC6959063

[B21] Djamgoz M. B. A. (2025). Stemness of cancer: a study of triple-negative breast cancer from a neuroscience perspective. Stem Cell. Rev. Rep. 21 (2), 337–350. 10.1007/s12015-024-10809-0 39531198 PMC11872763

[B22] Dong Y. Tu R. Liu H. Qing G. (2020). Regulation of cancer cell metabolism: oncogenic MYC in the driver's seat. Signal Transduct. Target Ther. 5 (1), 124. 10.1038/s41392-020-00235-2 32651356 PMC7351732

[B23] Ewen M. E. Oliver C. J. Sluss H. K. Miller S. J. Peeper D. S. (1995). p53-dependent repression of CDK4 translation in TGF-beta-induced G1 cell-cycle arrest. Genes. Dev. 9 (2), 204–217. 10.1101/gad.9.2.204 7851794

[B24] Ferralli J. Chiquet-Ehrismann R. Degen M. (2016). KLF4alpha stimulates breast cancer cell proliferation by acting as a KLF4 antagonist. Oncotarget 7 (29), 45608–45621. 10.18632/oncotarget.10058 27323810 PMC5216746

[B25] Fournier M. Javary J. Roh V. Fournier N. Radtke F. (2024). Reciprocal inhibition of NOTCH and SOX2 shapes tumor cell plasticity and therapeutic escape in triple-negative breast cancer. EMBO Mol. Med. 16 (12), 3184–3217. 10.1038/s44321-024-00161-8 39478150 PMC11628624

[B26] Fultang N. Chakraborty M. Peethambaran B. (2021). Regulation of cancer stem cells in triple negative breast cancer. Cancer Drug Resist 4 (2), 321–342. 10.20517/cdr.2020.106 35582030 PMC9019272

[B27] Giuli M. V. Giuliani E. Screpanti I. Bellavia D. Checquolo S. (2019). Notch signaling activation as a hallmark for triple-negative breast cancer subtype. J. Oncol. 2019, 8707053. 10.1155/2019/8707053 31379945 PMC6657611

[B28] Gorelik L. Flavell R. A. (2002). Transforming growth factor-beta in T-cell biology. Nat. Rev. Immunol. 2 (1), 46–53. 10.1038/nri704 11905837

[B29] Guo Z. Han S. (2023). Targeting cancer stem cell plasticity in triple-negative breast cancer. Explor Target Antitumor Ther. 4 (6), 1165–1181. 10.37349/etat.2023.00190 38213533 PMC10776602

[B30] Guo P. Chen W. Li H. Li M. Li L. (2018). The histone acetylation modifications of breast cancer and their therapeutic implications. Pathol. Oncol. Res. 24 (4), 807–813. 10.1007/s12253-018-0433-5 29948617

[B31] Gwak J. M. Kim M. Kim H. J. Jang M. H. Park S. Y. (2017). Expression of embryonal stem cell transcription factors in breast cancer: Oct4 as an indicator for poor clinical outcome and tamoxifen resistance. Oncotarget 8 (22), 36305–36318. 10.18632/oncotarget.16750 28422735 PMC5482656

[B32] Habib J. G. O'Shaughnessy J. A. (2016). The hedgehog pathway in triple-negative breast cancer. Cancer Med. 5 (10), 2989–3006. 10.1002/cam4.833 27539549 PMC5083752

[B33] Hanahan D. (2026). Hallmarks of cancer-then and now, and beyond. Cell. 189, 1–24. 10.1016/j.cell.2025.12.049 41616779

[B34] Haq A. T. A. Yang P. P. Jin C. Shih J. H. Chen L. M. Tseng H. Y. (2024). Immunotherapeutic IL-6R and targeting the MCT-1/IL-6/CXCL7/PD-L1 circuit prevent relapse and metastasis of triple-negative breast cancer. Theranostics 14 (5), 2167–2189. 10.7150/thno.92922 38505617 PMC10945351

[B35] Hassiotou F. Beltran A. Chetwynd E. Stuebe A. M. Twigger A. J. Metzger P. (2012). Breastmilk is a novel source of stem cells with multilineage differentiation potential. Stem Cells 30 (10), 2164–2174. 10.1002/stem.1188 22865647 PMC3468727

[B36] Hassiotou F. Hepworth A. R. Beltran A. S. Mathews M. M. Stuebe A. M. Hartmann P. E. (2013). Expression of the pluripotency transcription factor OCT4 in the normal and aberrant mammary gland. Front. Oncol. 3, 79. 10.3389/fonc.2013.00079 23596564 PMC3622876

[B37] He L. Wick N. Germans S. K. Peng Y. (2021). The role of breast cancer stem cells in chemoresistance and metastasis in triple-negative breast cancer. Cancers (Basel) 13 (24), 6209. 10.3390/cancers13246209 34944829 PMC8699562

[B38] He Q. Hu H. Yang F. Song D. Zhang X. Dai X. (2023). Advances in chimeric antigen receptor T cells therapy in the treatment of breast cancer. Biomed. Pharmacother. 162, 114609. 10.1016/j.biopha.2023.114609 37001182

[B39] Horiuchi D. Kusdra L. Huskey N. E. Chandriani S. Lenburg M. E. Gonzalez-Angulo A. M. (2012). MYC pathway activation in triple-negative breast cancer is synthetic lethal with CDK inhibition. J. Exp. Med. 209 (4), 679–696. 10.1084/jem.20111512 22430491 PMC3328367

[B40] Hossain F. Sorrentino C. Ucar D. A. Peng Y. Matossian M. Wyczechowska D. (2018). Notch signaling regulates mitochondrial metabolism and NF-kappaB activity in triple-negative breast cancer cells via IKKalpha-dependent non-canonical pathways. Front. Oncol. 8, 575. 10.3389/fonc.2018.00575 30564555 PMC6289043

[B41] Hu X. Zhang Q. Xing W. Wang W. (2022). Role of microRNA/lncRNA intertwined with the Wnt/beta-Catenin axis in regulating the pathogenesis of triple-negative breast cancer. Front. Pharmacol. 13, 814971. 10.3389/fphar.2022.814971 35814205 PMC9263262

[B42] Huang P. Zhou X. Zheng M. Yu Y. Jin G. Zhang S. (2023). Regulatory T cells are associated with the tumor immune microenvironment and immunotherapy response in triple-negative breast cancer. Front. Immunol. 14, 1263537. 10.3389/fimmu.2023.1263537 37767092 PMC10521732

[B43] Jaiswal A. Murakami K. Elia A. Shibahara Y. Done S. J. Wood S. A. (2021). Therapeutic inhibition of USP9x-mediated notch signaling in triple-negative breast cancer. Proc. Natl. Acad. Sci. U. S. A. 118 (38), e2101592118. 10.1073/pnas.2101592118 34518219 PMC8463885

[B44] Ji X. Naito Y. Weng H. Ma X. Endo K. Kito N. (2013). Renoprotective mechanisms of pirfenidone in hypertension-induced renal injury: through anti-fibrotic and anti-oxidative stress pathways. Biomed. Res. 34 (6), 309–319. 10.2220/biomedres.34.309 24389407

[B45] Katoh M. Katoh M. (2022). WNT signaling and cancer stemness. Essays Biochem. 66 (4), 319–331. 10.1042/EBC20220016 35837811 PMC9484141

[B46] Katsuta E. Yan L. Takeshita T. McDonald K. A. Dasgupta S. Opyrchal M. (2019). High MYC mRNA expression is more clinically relevant than MYC DNA amplification in triple-negative breast cancer. Int. J. Mol. Sci. 21 (1), 217. 10.3390/ijms21010217 31905596 PMC6981812

[B47] Kausar M. A. Anwar S. El-Horany H. E. S. Khan F. H. Tyagi N. Najm M. Z. (2023). Journey of CAR T-cells: emphasising the concepts and advancements in breast cancer (Review). Int. J. Oncol. 63 (6), 130. 10.3892/ijo.2023.5578 37830150 PMC10622179

[B48] Kim S. Y. Kang J. W. Song X. Kim B. K. Yoo Y. D. Kwon Y. T. (2013). Role of the IL-6-JAK1-STAT3-Oct-4 pathway in the conversion of non-stem cancer cells into cancer stem-like cells. Cell. Signal 25 (4), 961–969. 10.1016/j.cellsig.2013.01.007 23333246 PMC3595341

[B49] Kim J. Y. Kim J. C. Lee J. Y. Park M. J. (2018). Oct4 suppresses IR-induced premature senescence in breast cancer cells through STAT3- and NF-kappaB-mediated IL-24 production. Int. J. Oncol. 53 (1), 47–58. 10.3892/ijo.2018.4391 29749438 PMC5958730

[B50] King T. D. Suto M. J. Li Y. (2012). The Wnt/Beta-catenin signaling pathway: a potential therapeutic target in the treatment of triple negative breast cancer. J. Cell. Biochem. 113 (1), 13–18. 10.1002/jcb.23350 21898546 PMC10924801

[B51] Klauber-DeMore N. Schulte B. A. Wang G. Y. (2018). Targeting MYC for triple-negative breast cancer treatment. Oncoscience 5 (5-6), 120–121. 10.18632/oncoscience.414 30035158 PMC6049315

[B52] Kontomanolis E. N. Kalagasidou S. Pouliliou S. Anthoulaki X. Georgiou N. Papamanolis V. (2018). The notch pathway in breast cancer progression. Sci. World J. 2018, 2415489. 10.1155/2018/2415489 30111989 PMC6077551

[B53] Lee J. V. Housley F. Yau C. Nakagawa R. Winkler J. Anttila J. M. (2022). Combinatorial immunotherapies overcome MYC-driven immune evasion in triple negative breast cancer. Nat. Commun. 13 (1), 3671. 10.1038/s41467-022-31238-y 35760778 PMC9237085

[B54] Leis O. Eguiara A. Lopez-Arribillaga E. Alberdi M. J. Hernandez-Garcia S. Elorriaga K. (2012). Sox2 expression in breast tumours and activation in breast cancer stem cells. Oncogene 31 (11), 1354–1365. 10.1038/onc.2011.338 21822303

[B55] Li X. Xu Y. Chen Y. Chen S. Jia X. Sun T. (2013). SOX2 promotes tumor metastasis by stimulating epithelial-to-mesenchymal transition via regulation of WNT/beta-catenin signal network. Cancer Lett. 336 (2), 379–389. 10.1016/j.canlet.2013.03.027 23545177

[B56] Li L. Tang P. Li S. Qin X. Yang H. Wu C. (2017). Notch signaling pathway networks in cancer metastasis: a new target for cancer therapy. Med. Oncol. 34 (10), 180. 10.1007/s12032-017-1039-6 28918490

[B57] Li X. Xiang Y. Li F. Yin C. Li B. Ke X. (2019). WNT/beta-Catenin signaling pathway regulating T cell-inflammation in the tumor microenvironment. Front. Immunol. 10, 2293. 10.3389/fimmu.2019.02293 31616443 PMC6775198

[B58] Liu C. G. Lu Y. Wang B. B. Zhang Y. J. Zhang R. S. Lu Y. (2011). Clinical implications of stem cell gene Oct-4 expression in breast cancer. Ann. Surg. 253 (6), 1165–1171. 10.1097/SLA.0b013e318214c54e 21394007

[B59] Liu P. Tang H. Song C. Wang J. Chen B. Huang X. (2018). SOX2 promotes cell proliferation and metastasis in triple negative breast cancer. Front. Pharmacol. 9, 942. 10.3389/fphar.2018.00942 30186173 PMC6110877

[B60] Lu X. Mazur S. J. Lin T. Appella E. Xu Y. (2014). The pluripotency factor nanog promotes breast cancer tumorigenesis and metastasis. Oncogene 33 (20), 2655–2664. 10.1038/onc.2013.209 23770853 PMC3925756

[B61] Luke J. J. Bao R. Sweis R. F. Spranger S. Gajewski T. F. (2019). WNT/beta-catenin pathway activation correlates with immune exclusion across human cancers. Clin. Cancer Res. 25 (10), 3074–3083. 10.1158/1078-0432.CCR-18-1942 30635339 PMC6522301

[B62] Mahanujam A. (2025). The role of beta-catenin and the Wnt signalling pathway in breast cancer initiation, progression and metastasis: a literature review. URNCST J. 9 (1–11). 10.26685/urncst.715

[B63] Mahendran G. Shangaradas A. D. Romero-Moreno R. Wickramarachchige Dona N. Sarasija S. H. G. S. Perera S. (2024). Unlocking the epigenetic code: new insights into triple-negative breast cancer. Front. Oncol. 14, 1499950. 10.3389/fonc.2024.1499950 39744000 PMC11688480

[B64] McAuliffe S. M. Morgan S. L. Wyant G. A. Tran L. T. Muto K. W. Chen Y. S. (2012). Targeting notch, a key pathway for ovarian cancer stem cells, sensitizes tumors to platinum therapy. Proc. Natl. Acad. Sci. U. S. A. 109 (43), E2939–E2948. 10.1073/pnas.1206400109 23019585 PMC3491453

[B65] Mediratta K. El-Sahli S. D’Costa V. Wang L. (2020). Current progresses and challenges of immunotherapy in triple-negative breast cancer. Cancers (Basel) 12 (12), 3529. 10.3390/cancers12123529 33256070 PMC7761500

[B66] Merikhian P. Eisavand M. R. Farahmand L. (2021). Triple-negative breast cancer: understanding Wnt signaling in drug resistance. Cancer Cell. Int. 21 (1), 419. 10.1186/s12935-021-02107-3 34376211 PMC8353874

[B67] Mir P. A. Kumar N. Gupta S. K. Kaur A. Farooq S. Sandhu G. S. (2025). Therapeutic innovations in triple negative breast cancer: integrating molecular targeting and monoclonal antibody strategies. Front. Oncol. 15, 1645438. 10.3389/fonc.2025.1645438 41069341 PMC12504885

[B68] Mou W. Xu Y. Ye Y. Chen S. Li X. Gong K. (2015). Expression of Sox2 in breast cancer cells promotes the recruitment of M2 macrophages to tumor microenvironment. Cancer Lett. 358 (2), 115–123. 10.1016/j.canlet.2014.11.004 25444903

[B69] Mukherjee P. Gupta A. Chattopadhyay D. Chatterji U. (2017). Modulation of SOX2 expression delineates an end-point for paclitaxel-effectiveness in breast cancer stem cells. Sci. Rep. 7 (1), 9170. 10.1038/s41598-017-08971-2 28835684 PMC5569040

[B70] Mulkearns-Hubert E. E. Esakov Rhoades E. Ben-Salem S. Bharti R. Hajdari N. Johnson S. (2024). Targeting NANOG and FAK via Cx26-derived cell-penetrating peptides in triple-negative breast cancer. Mol. Cancer Ther. 23 (1), 56–67. 10.1158/1535-7163.MCT-21-0783 37703580 PMC10840808

[B71] Nagata T. Shimada K. Long L. X. Okumura T. Tazawa K. Higashiyama K. (2016). KLF4 improve prognosis of triple-negative breast cancer by suppression of epithelial-mesenchymal transition. Breast Cancer Res. 1 (110), 2. 10.4172/2572-4118.1000110

[B72] Nagata T. Shimada Y. Sekine S. Moriyama M. Hashimoto I. Matsui K. (2017). KLF4 and NANOG are prognostic biomarkers for triple-negative breast cancer. Breast Cancer 24 (2), 326–335. 10.1007/s12282-016-0708-1 27300169

[B73] Noh K. H. Kim B. W. Song K. H. Cho H. Lee Y. H. Kim J. H. (2012). Nanog signaling in cancer promotes stem-like phenotype and immune evasion. J. Clin. Invest. 122 (11), 4077–4093. 10.1172/JCI64057 23093782 PMC3484451

[B74] Noman A. S. Uddin M. Rahman M. Z. Nayeem M. J. Alam S. S. Khatun Z. (2016). Overexpression of sonic hedgehog in the triple negative breast cancer: clinicopathological characteristics of high burden breast cancer patients from Bangladesh. Sci. Rep. 6, 18830. 10.1038/srep18830 26727947 PMC4700415

[B75] Okuda H. Xing F. Pandey P. R. Sharma S. Watabe M. Pai S. K. (2013). miR-7 suppresses brain metastasis of breast cancer stem-like cells by modulating KLF4. Cancer Res. 73 (4), 1434–1444. 10.1158/0008-5472.CAN-12-2037 23384942 PMC3576138

[B76] Pacella I. Cammarata I. Focaccetti C. Miacci S. Gulino A. Tripodo C. (2018). Wnt3a neutralization enhances T-cell responses through indirect mechanisms and restrains tumor growth. Cancer Immunol. Res. 6 (8), 953–964. 10.1158/2326-6066.CIR-17-0713 30018042

[B77] Pajcini K. V. Speck N. A. Pear W. S. (2011). Notch signaling in mammalian hematopoietic stem cells. Leukemia 25 (10), 1525–1532. 10.1038/leu.2011.127 21647159 PMC5924479

[B78] Palla M. Scarpato L. Di Trolio R. Ascierto P. A. (2022). Sonic hedgehog pathway for the treatment of inflammatory diseases: implications and opportunities for future research. J. Immunother. Cancer 10 (6), e004397. 10.1136/jitc-2021-004397 35710292 PMC9204405

[B79] Pandya A. Y. Talley L. I. Frost A. R. Fitzgerald T. J. Trivedi V. Chakravarthy M. (2004). Nuclear localization of KLF4 is associated with an aggressive phenotype in early-stage breast cancer. Clin. Cancer Res. 10 (8), 2709–2719. 10.1158/1078-0432.ccr-03-0484 15102675

[B80] Pardali K. Moustakas A. (2007). Actions of TGF-beta as tumor suppressor and pro-metastatic factor in human cancer. Biochim. Biophys. Acta 1775 (1), 21–62. 10.1016/j.bbcan.2006.06.004 16904831

[B81] Pardo I. Fagundes P. B. Oliveira R. S. d. Campregher P. V. (2024). A molecular approach to triple-negative breast cancer: targeting the Notch signaling pathway. Einstein (Sao Paulo) 22, eRW0552. 10.31744/einstein_journal/2024RW0552 38324848 PMC10948095

[B82] Park S. Y. Choi J. H. Nam J. S. (2019). Targeting cancer stem cells in triple-negative breast cancer. Cancers (Basel) 11 (7), 965. 10.3390/cancers11070965 31324052 PMC6678244

[B83] Patel S. Alam A. Pant R. Chattopadhyay S. (2019). Wnt signaling and its significance within the tumor microenvironment: novel therapeutic insights. Front. Immunol. 10, 2872. 10.3389/fimmu.2019.02872 31921137 PMC6927425

[B84] Phi L. T. H. Sari I. N. Yang Y. G. Lee S. H. Jun N. Kim K. S. (2018). Cancer stem cells (CSCs) in drug resistance and their therapeutic implications in cancer treatment. Stem Cells Int. 2018, 5416923. 10.1155/2018/5416923 29681949 PMC5850899

[B85] Pratelli G. Carlisi D. Di Liberto D. Notaro A. Giuliano M. D'Anneo A. (2023). MCL1 inhibition overcomes the aggressiveness features of triple-negative breast cancer MDA-MB-231 cells. Int. J. Mol. Sci. 24 (13), 11149. 10.3390/ijms241311149 37446326 PMC10342057

[B86] Quatromoni J. G. Suzuki E. Okusanya O. Judy B. F. Bhojnagarwala P. Venegas O. (2013). The timing of TGF-beta inhibition affects the generation of antigen-specific CD8+ T cells. BMC Immunol. 14, 30. 10.1186/1471-2172-14-30 23865808 PMC3725164

[B87] Ray A. Alalem M. Ray B. K. (2013). Loss of epigenetic Kruppel-like factor 4 histone deacetylase (KLF-4-HDAC)-mediated transcriptional suppression is crucial in increasing vascular endothelial growth factor (VEGF) expression in breast cancer. J. Biol. Chem. 288 (38), 27232–27242. 10.1074/jbc.M113.481184 23926105 PMC3779720

[B88] Reis-Filho J. S. Tutt A. N. (2008). Triple negative tumours: a critical review. Histopathology 52 (1), 108–118. 10.1111/j.1365-2559.2007.02889.x 18171422

[B89] Riobo-Del Galdo N. A. Lara Montero A. Wertheimer E. V. (2019). Role of Hedgehog signaling in breast cancer: pathogenesis and therapeutics. Cells 8 (4), 375. 10.3390/cells8040375 31027259 PMC6523618

[B90] Roberts M. S. Anstine L. J. Finke V. S. Bryson B. L. Webb B. M. Weber-Bonk K. L. (2020). KLF4 defines the efficacy of the epidermal growth factor receptor inhibitor, erlotinib, in triple-negative breast cancer cells by repressing the EGFR gene. Breast Cancer Res. 22 (1), 66. 10.1186/s13058-020-01305-7 32552913 PMC7301986

[B91] Rogaczewski P. Janiak M. Borysewicz K. Głos T. Ahmadi N. Szylberg Ł. (2022). Clinical significancy of WNT pathway inhibition in various cancers. J. Educ. Health Sport 12 (11), 183–191. 10.12775/jehs.2022.12.11.024

[B92] Romaniuk-Drapala A. Totoń E. Taube M. Idzik M. Rubiś B. Lisiak N. (2024). Breast cancer stem cells and tumor heterogeneity: characteristics and therapeutic strategies. Cancers (Basel) 16 (13), 2481. 10.3390/cancers16132481 39001543 PMC11240630

[B93] Rosado-Sanz M. Martínez-Alarcón N. Abellán-Soriano A. Golfe R. Trinidad E. M. Font de Mora J. (2025). Cytokine networks in triple-negative breast cancer: mechanisms, therapeutic targets, and emerging strategies. Biomedicines 13 (8), 1945. 10.3390/biomedicines13081945 40868199 PMC12383849

[B94] Saltis J. (1996). TGF-beta: receptors and cell cycle arrest. Mol. Cell. Endocrinol. 116 (2), 227–232. 10.1016/0303-7207(95)03721-7 8647324

[B95] Samanta S. Sun H. Goel H. L. Pursell B. Chang C. Khan A. (2016). IMP3 promotes stem-like properties in triple-negative breast cancer by regulating SLUG. Oncogene 35 (9), 1111–1121. 10.1038/onc.2015.164 25982283 PMC5784260

[B96] Sardesai S. Badawi M. Mrozek E. Morgan E. Phelps M. Stephens J. (2020). A phase I study of an oral selective gamma secretase (GS) inhibitor RO4929097 in combination with neoadjuvant paclitaxel and carboplatin in triple negative breast cancer. Invest. New Drugs 38 (5), 1400–1410. 10.1007/s10637-020-00895-5 31953695 PMC7955776

[B97] Schott A. F. Landis M. D. Dontu G. Griffith K. A. Layman R. M. Krop I. (2013). Preclinical and clinical studies of gamma secretase inhibitors with docetaxel on human breast tumors. Clin. Cancer Res. 19 (6), 1512–1524. 10.1158/1078-0432.CCR-11-3326 23340294 PMC3602220

[B98] Sen U. Shanavas S. Nagendra A. H. Nihad M. Chaudhury D. Rachamalla H. K. (2023). Significance of Oct-4 transcription factor as a pivotal therapeutic target for CD44(+)/24(-) mammary tumor initiating cells: aiming at the root of the recurrence. Cell. Biol. Int. 47 (4), 742–753. 10.1002/cbin.11978 36573403

[B99] Serrano Garcia L. Jávega B. Llombart Cussac A. Gión M. Pérez-García J. M. Cortés J. (2024). Patterns of immune evasion in triple-negative breast cancer and new potential therapeutic targets: a review. Front. Immunol. 15, 1513421. 10.3389/fimmu.2024.1513421 39735530 PMC11671371

[B100] Siegel P. M. Massague J. (2003). Cytostatic and apoptotic actions of TGF-beta in homeostasis and cancer. Nat. Rev. Cancer 3 (11), 807–821. 10.1038/nrc1208 14557817

[B101] Sriramulu S. Thoidingjam S. Speers C. Nyati S. (2024). Present and future of immunotherapy for triple-negative breast cancer. Cancers (Basel) 16 (19), 3250. 10.3390/cancers16193250 39409871 PMC11475478

[B102] Takai K. Le A. Weaver V. M. Werb Z. (2016). Targeting the cancer-associated fibroblasts as a treatment in triple-negative breast cancer. Oncotarget 7 (50), 82889–82901. 10.18632/oncotarget.12658 27756881 PMC5341254

[B103] Takebe N. Harris P. J. Warren R. Q. Ivy S. P. (2011). Targeting cancer stem cells by inhibiting Wnt, Notch, and Hedgehog pathways. Nat. Rev. Clin. Oncol. 8 (2), 97–106. 10.1038/nrclinonc.2010.196 21151206

[B104] Taki M. Abiko K. Ukita M. Murakami R. Yamanoi K. Yamaguchi K. (2021). Tumor immune microenvironment during epithelial-mesenchymal transition. Clin. Cancer Res. 27 (17), 4669–4679. 10.1158/1078-0432.CCR-20-4459 33827891

[B105] Tang M. O’Grady S. Crown J. Duffy M. J. (2022). MYC as a therapeutic target for the treatment of triple-negative breast cancer: preclinical investigations with the novel MYC inhibitor, MYCi975. Breast Cancer Res. Treat. 195 (2), 105–115. 10.1007/s10549-022-06673-6 35908121 PMC9374613

[B106] Thiagarajan P. S. Hitomi M. Hale J. S. Alvarado A. G. Otvos B. Sinyuk M. (2015). Development of a fluorescent reporter system to delineate cancer stem cells in triple-negative breast cancer. Stem Cells 33 (7), 2114–2125. 10.1002/stem.2021 25827713 PMC4494654

[B107] Valent P. Bonnet D. De Maria R. Lapidot T. Copland M. Melo J. V. (2012). Cancer stem cell definitions and terminology: the devil is in the details. Nat. Rev. Cancer 12 (11), 767–775. 10.1038/nrc3368 23051844

[B108] Wan Y. Y. Flavell R. A. (2007). Yin-Yang' functions of transforming growth factor-beta and T regulatory cells in immune regulation. Immunol. Rev. 220, 199–213. 10.1111/j.1600-065X.2007.00565.x 17979848 PMC2614905

[B109] Wang M. L. Chiou S. H. Wu C. W. (2013). Targeting cancer stem cells: emerging role of Nanog transcription factor. Onco Targets Ther. 6, 1207–1220. 10.2147/OTT.S38114 24043946 PMC3772775

[B110] Wang B. Zhao M. Z. Cui N. P. Lin D. D. Zhang A. Y. Qin Y. (2015). Kruppel-like factor 4 induces apoptosis and inhibits tumorigenic progression in SK-BR-3 breast cancer cells. FEBS Open Bio 5, 147–154. 10.1016/j.fob.2015.02.003 25834779 PMC4359971

[B111] Wang X. Han Y. Peng J. He J. (2021). CCR5 is a prognostic biomarker and an immune regulator for triple negative breast cancer. Aging (Albany NY) 13 (20), 23810–23830. 10.18632/aging.203654 34717291 PMC8580338

[B112] Won K. A. Spruck C. (2020). Triple-negative breast cancer therapy: current and future perspectives (review). Int. J. Oncol. 57 (6), 1245–1261. 10.3892/ijo.2020.5135 33174058 PMC7646583

[B113] Wu S. Y. Xiao Y. Wei J. L. Xu X. E. Jin X. Hu X. (2021). MYC suppresses STING-dependent innate immunity by transcriptionally upregulating DNMT1 in triple-negative breast cancer. J. Immunother. Cancer 9 (7), e002528. 10.1136/jitc-2021-002528 34321275 PMC8320259

[B114] Yang A. Qin S. Schulte B. A. Ethier S. P. Tew K. D. Wang G. Y. (2017). MYC inhibition depletes cancer stem-like cells in triple-negative breast cancer. Cancer Res. 77 (23), 6641–6650. 10.1158/0008-5472.CAN-16-3452 28951456 PMC5712265

[B115] Yin L. Duan J. J. Bian X. W. Yu S. C. (2020). Triple-negative breast cancer molecular subtyping and treatment progress. Breast Cancer Res. 22 (1), 61. 10.1186/s13058-020-01296-5 32517735 PMC7285581

[B116] Zeng Z. Fu M. Hu Y. Wei Y. Wei X. Luo M. (2023). Regulation and signaling pathways in cancer stem cells: implications for targeted therapy for cancer. Mol. Cancer 22 (1), 172. 10.1186/s12943-023-01877-w 37853437 PMC10583419

[B117] Zhang Y. Wang X. (2020). Targeting the Wnt/beta-catenin signaling pathway in cancer. J. Hematol. Oncol. 13 (1), 165. 10.1186/s13045-020-00990-3 33276800 PMC7716495

[B118] Zhang C. Samanta D. Lu H. Bullen J. W. Zhang H. Chen I. (2016). Hypoxia induces the breast cancer stem cell phenotype by HIF-dependent and ALKBH5-mediated m(6)A-demethylation of NANOG mRNA. Proc. Natl. Acad. Sci. U. S. A. 113 (14), E2047–E2056. 10.1073/pnas.1602883113 27001847 PMC4833258

[B119] Zhang Q. Han Z. Zhu Y. Chen J. Li W. (2020). The role and specific mechanism of OCT4 in cancer stem cells: a review. Int. J. Stem Cells 13 (3), 312–325. 10.15283/ijsc20097 32840233 PMC7691851

[B120] Zheng M. Bocangel D. Doneske B. Mhashilkar A. Ramesh R. Hunt K. K. (2007). Human interleukin 24 (MDA-7/IL-24) protein kills breast cancer cells via the IL-20 receptor and is antagonized by IL-10. Cancer Immunol. Immunother. 56 (2), 205–215. 10.1007/s00262-006-0175-1 16710719 PMC11030656

[B121] Zheng Y. Li S. Tang H. Meng X. Zheng Q. (2023). Molecular mechanisms of immunotherapy resistance in triple-negative breast cancer. Front. Immunol. 14, 1153990. 10.3389/fimmu.2023.1153990 37426654 PMC10327275

[B122] Zhu H. Gu X. Xia L. Zhou Y. Bouamar H. Yang J. (2018). A novel TGFbeta trap blocks chemotherapeutics-induced TGFbeta1 signaling and enhances their anticancer activity in gynecologic cancers. Clin. Cancer Res. 24 (12), 2780–2793. 10.1158/1078-0432.CCR-17-3112 29549162 PMC6004245

[B123] Zhu X. Wang K. Chen Y. (2020). Ophiopogonin D suppresses TGF-beta1-mediated metastatic behavior of MDA-MB-231 breast carcinoma cells via regulating ITGB1/FAK/Src/AKT/beta-catenin/MMP-9 signaling axis. Toxicol Vitro 69, 104973. 10.1016/j.tiv.2020.104973 32818624

[B124] Zou H. Chen H. Zhou Z. Wan Y. Liu Z. (2019). ATXN3 promotes breast cancer metastasis by deubiquitinating KLF4. Cancer Lett. 467, 19–28. 10.1016/j.canlet.2019.09.012 31563563

